# Acute exposure to 27.12 MHz radiofrequency electromagnetic field disrupts blood-brain barrier integrity via eNOS activation and occludin down-regulation

**DOI:** 10.22038/ijbms.2025.86952.18784

**Published:** 2025

**Authors:** Arzu Ulusoy, Halil Asci, Rumeysa Taner, Muhammet Yusuf Tepebasi, Ilter Ilhan, Pinar Karabacak, Selcuk Comlekci, Ozlem Ozmen

**Affiliations:** 1 Department of Bioengineering, Institute of Natural and Applied Sciences, Suleyman Demirel University, Isparta, Türkiye; 2 Department of Pharmacology, Faculty of Medicine, Suleyman Demirel University, Isparta, Türkiye; 3 Department of Genetics, Faculty of Medicine, Suleyman Demirel University, Isparta, Türkiye; 4 Department of Biochemistry, Faculty of Medicine, Suleyman Demirel University, Isparta, Türkiye; 5 Department of Anesthesia and Reanimation, Faculty of Medicine, Suleyman Demirel University, Isparta, Türkiye; 6 Department of Electrical and Electronic Engineering, Suleyman Demirel University, Isparta, Türkiye; 7 Department of Pathology, Faculty of Veterinary Medicine, Burdur Mehmet Akif Ersoy University, Burdur, Türkiye

**Keywords:** Blood-Brain Barrier, CD31, Claudin-1, eNOS, Haptoglobin, Occludin, Radiofrequency- electromagnetic fields

## Abstract

**Objective(s):**

Controlled modulation of blood-brain barrier (BBB) permeability without inducing damage represents a promising strategy for enhancing central nervous system drug delivery.

**Materials and Methods:**

This study investigated the effects of 27.12 MHz radiofrequency electromagnetic field (RF-EMF) exposure for 30, 180, and 360 min on BBB integrity in female Wistar rats (n=6 per group). Brain and cerebellar tissues were analyzed histologically and immunohistochemically (haptoglobin, CD31), and molecular analyses were performed for oxidative stress parameters (TAS, TOS, and OSI), inflammatory and hypoxia markers (IL-6 and HIF-1α), endothelial nitric oxide synthase (eNOS), and tight junction proteins (claudin-1 and occludin).

**Results:**

RF-EMF exposure for 30 min significantly increased eNOS gene expression without triggering oxidative stress or inflammation. Longer exposures (180 and 360 min) resulted in increased HIF-1α expression, decreased levels of claudin-1 and occludin, and histopathological signs of hyperemia and edema, particularly in the cerebellar tissue. Notably, occludin expression decreased in all exposure groups, while eNOS was maximally up-regulated at 30 min.

**Conclusion:**

Thirty-minute RF-EMF exposure at 27.12 MHz transiently modulates BBB permeability via eNOS activation and occludin down-regulation without apparent tissue damage. These findings suggest that short-term RF-EMF application may serve as a non-invasive tool for targeted BBB modulation. Further studies are warranted to refine exposure parameters and assess translational potential in neuropharmacological applications.

## Introduction

The blood-brain barrier (BBB) is a dynamic interface that protects the central nervous system by regulating the passage of substances between the bloodstream and brain parenchyma. While it restricts harmful molecules, it also limits therapeutic drug access to brain tissues, posing a significant obstacle in treating neurological diseases (1, 2).

Modulating BBB permeability in a controlled, reversible, and non-damaging manner is of high clinical interest. Such modulation could allow for enhanced delivery of drugs, including anesthetics, at lower doses, potentially reducing systemic side effects (3, 4). The integrity of the BBB is tightly maintained by tight junction proteins such as occludin and claudins, as well as metabolic enzymes and transporters. Disruption of these structures is often associated with neuroinflammation and oxidative stress (5, 6).

The passage of substances through the BBB is tightly controlled both physically by tight junctions (TJs) and metabolically by a barrier formed by various enzymes (6). It is known that the permeability of the BBB increases with decreased levels of structures such as claudin-5, occludin-1, and zonulin-1, which provide information about the state of the TJ. Studies are showing that this permeability may also increase in various inflammatory events due to damage (5, 6). 

Haptoglobin, an acute-phase response protein, neutralizes hemoglobin in the bloodstream and defends neurons from damage induced by hemolytic products (7). Platelet endothelial cell adhesion molecule-1 (PECAM-1; CD31) regulates vascular repair mechanisms to restore BBB integrity (8, 9).

Increased levels of interleukin-6 (IL-6), which is also an acute phase reactant, and increased expression of hypoxia-inducible factor-1 alpha (HIF-1α) in the presence of hypoxia, regulate inflammatory responses and increase BBB permeability by increasing eNOS-mediated NO synthesis (10, 11).

In a review study examining the effect of NO on BBB integrity, Thiel and Audus (2001) stated that since the BBB is composed of endothelial cells lining the capillaries of the brain, NO will cause the BBB to open under many conditions, including NO-mediated transients in the transmission of substances, which may eventually cause vasogenic edema and secondary brain damage (12).

Radiofrequency electromagnetic field (RF-EMF) is a non-invasive, non-contact adjunctive therapy that involves the delivery of an RF-EMF to a target tissue without direct electrode contact with the body, using a carrier frequency of 27.12 MHz, the medical frequency set by the Federal Communications Commission (13). Bragin *et al*. (2015) demonstrated that a 30-minute RF-EMF exposure at 27.12 MHz increased cerebral arteriolar dilation and NO release, suggesting a vasogenic effect without tissue injury (14). In a recent preliminary study, Taner *et al*. (2023) observed synergistic effects of RF and pulsed magnetic fields on ischemia-reperfusion-induced brain injury, with enhanced perfusion and reduced infarct size (15).

In this study, the effect of 27.12 MHz RF-EMF applied to experimental animals for different durations on the BBB and the NO connection was investigated.

## Materials and Methods

### Animals and ethical approval

All the experimental procedures were performed under the guidelines of the Animal Research: Reporting *in vivo* Experiments (ARRIVE) guidelines 2.0 and were approved by the Committee on Animal Research of Suleyman Demirel University, Isparta (Approval No: 26.01.2023/01-129). Twenty-four adult Wistar Albino female rats, weighing 300-350 g, were obtained from the Suleyman Demirel University Experimental Animal Research Laboratory and housed at 21-22 ^°^C and 60%±5% humidity, with a 12-hour light:12-hour dark cycle. They were fed standard commercial feed ad libitum and provided with water during the experiments. Additionally, Suleyman Demirel University’s Scientific Research Project Unit (SDU-BAP) provided financial support for the study (Project No.: TSG-2024-9515).

Only female rats were used to minimize the confounding influence of androgen-driven vascular responses and to ensure hormonal consistency across groups, given the potential sex differences in endothelial nitric oxide synthesis and BBB permeability.

### RF-EMF application

**Figure 1:**
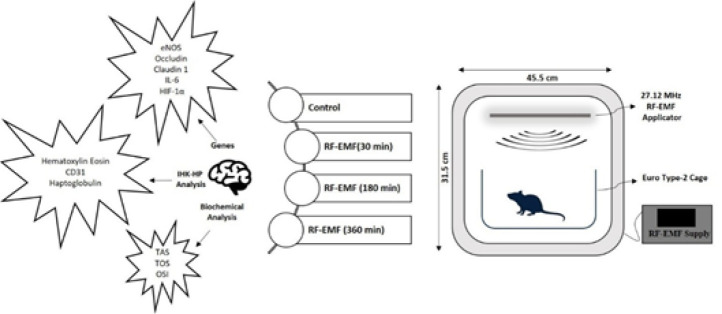
Study design and RF-EMF (27.12 MHz) exposure setup for Wistar Albino rats

As it is known, 27.12 MHz is located in the part of the ISM Band reserved for Medical Applications. In this study, a quartz crystal oscillator circuit operating at 27.12 MHz, with a power transistor amplifier amplifying the oscillator output, was used. The high-frequency electromagnetic field was designed for homogeneous exposure by ensuring that the exposure was uniform at every point and that the animals could move freely in the plexiglass cages (EuroType-2) where they were placed. In addition, considering the wavelength at the propagation frequency (11 m), each point of the cage exposure device is within the near-field region of the source, so a near-field probe can be suitable for observing the frequency used as an antenna at every stage of the experiments and measurements. As is known, the wavelength for this frequency is approximately 11 meters, and the near-field and far-field limits are 1.75 meters. In other words, all experiments have been conducted in the near field region. The electric field value was targeted as 10 V/m. This value was easily obtained with an additional transistor oscillator within acceptable limits (Figure 1).

The shielding efficiency of the electromagnetic isolation room (Faraday Cage) in the laboratory was determined to be 80 dB. For the working frequency of 27.12 MHz, and to minimize the interference effects of possible repetitive reflections in the environment, spectrum analyzers (ROHDE-SCHWARZ FSH6, USA, and PROMAX AE-566 spectrum analyzer, USA) were used. RF-EMF measurements were conducted using a field meter (Mustool MT-525, China), an ELF meter (PF-4, UK), and an Extech 480836 (USA). The targeted 10 V/m electric field value for RF-EMF propagation was produced by the single-transistor oscillator we made as a custom design. The circuit design and antenna structure used in the RF-EMF setup are presented in Supplementary Figure 2.

**Figure 2:**
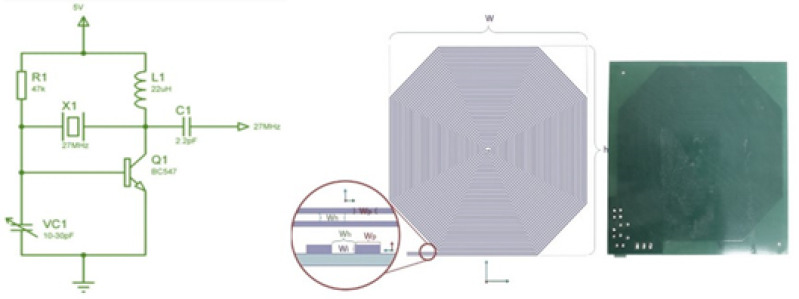
Circuit diagram and 27.12 MHz RF-EMF application antenna used for Wistar Albino rat exposure

As shown in Figure 2, the single-transistor oscillator also supplies energy to the antenna, serving as the applicator, and functions as a power amplifier. Since the circuit is connected directly to the antenna, with no antenna cable in between, the losses are minimal. In this case, two RF-EMF applicators, used 15 cm above the animal cages, provide approximately homogeneous RF-EMF radiation within the cage. An EMF meter was used to measure both the homogeneity within the cage and the obtained electric field value (EXTECH, EMF meter, FLIR Systems, Inc., USA)

The exposure durations (30, 180, and 360 min) were selected based on preliminary studies suggesting time-dependent nitric oxide dynamics and to evaluate both acute and prolonged RF-EMF effects on BBB physiology. The 30-minute duration was chosen in line with prior studies reporting peak eNOS activation at this interval (14), while longer durations aimed to explore cumulative or potentially adverse effects.”

### Experimental procedure

A total of 24 rats were divided into four groups after being obtained from Suleyman Demirel University Experimental Animal Laboratory. Groups were;

*Group 1 (Control)(n=6):* Rats were kept under the same conditions as the others, but there was no RF-EMF application. After 360 min (the longest RF-EMF application time), the rats were euthanized.

*Group 2 (30 min exposure)(n = 6):* Rats were exposed to RF-EMF for 30 min in the unit and then euthanized.

*Group 3 (180 min exposure)(n=6):* Rats were exposed to RF-EMF for 180 min in the unit and then euthanized.

*Group 4 (360 min exposure)(n=6):* Rats were exposed to RF-EMF for 360 min in the unit and then euthanized.

At the end of these applications, 90 mg/kg ketamine (Keta-Control, Doga Ilac, Turkey) and 8-10 mg/kg xylazine (Xylazinbio, Bioveta, Czech Republic) were given to animals and euthanized by the surgical exsanguination method following abdominal incision. The right hemispheres of the brain and cerebellum tissues were removed following the decapitation procedure and put into 10% formaldehyde for histopathological examination with hematoxylin-eosin staining and immunohistochemical analysis (CD31 and haptoglobin expressions) to examine tissue damage and increased permeability. The levels of the oxidative stress markers total antioxidant species (TAS), total oxidant status (TOS), oxidative stress index (OSI) biochemically, and the expressions of interleukin-6 (IL-6), hypoxia inducible factor-1 alpha (HIF-1α), endothelial nitric oxide synthase (eNOS), claudin-1, and occludin were investigated with RT-qPCR. 

From each group (n=6), three animals were allocated for histopathological and immunohistochemical analyses, while the remaining three were used for biochemical and genetic analyses (RT-qPCR, TAS, TOS, and OSI). This distribution ensured sufficient tissue availability for each method without compromising analytical integrity.

### Measurement of oxidative stress parameters in the brain tissue

Brain tissues of rats were homogenized with the Ultra Turrax Janke & Kunkel T-25 homogenizer (IKA® Werke, Germany) with 1:9 (w/v) phosphate-buffered saline (10 mM Na2HPO4, 1.8 mM KH2PO4, 2.7 mM KCl, 137 mM NaCl, pH 7.4). After that, homogenized liver tissues were analyzed spectrophotometrically for TAS, TOS, and OSI values, as previously described by Savran *et al*. (16). The TAS and TOS results in the serum were expressed in µmol Trolox Eq/L and µmol H2O2 Eq/L, respectively. The TAS and TOS results for the tissues were expressed as the ratio of these values to the protein value (17). The OSI was calculated using the formula OSI (TOS/TAS) x 10 (18). Protein levels of the supernatants were determined with a Beckman Coulter autoanalyzer (Beckman Coulter, USA). 

TAS levels reflect the cumulative antioxidant capacity of tissue, while TOS represents the total pro-oxidant burden. The OSI index, calculated as TOS/TAS×10, serves as a global indicator of oxidative stress status. All measurements were performed in duplicate using an automated colorimetric method developed by Erel (2004, 2005)(17, 18).

### Histopathological evaluation

During the necropsy, samples of the brain and cerebellum were carefully removed and preserved in 10% buffered formalin for histological analysis. After routinely processing the tissues, five-micrometer-thick sections of the paraffin blocks were cut using a rotary microtome (Leica RM2155, Leica Microsystems, Wetzlar, Germany). After deparaffinization, rehydration with ethanol of decreasing concentrations, staining with hematoxylin-eosin (HE), cleaning in xylene, and covering the sections, they were examined under a light microscope. Histological sections were evaluated at ×200 and ×400 magnifications using an Olympus BX53 microscope. Cortical and cerebellar regions were examined explicitly for vascular changes, edema, and neuronal damage. At the histopathological examination of the brain and cerebellum, hyperemia, hemorrhage, edema, gliosis, and neuronal damage were evaluated. 

All histopathological and immunohistochemical evaluations were performed in a blinded manner by two independent observers, who were unaware of the group allocations, to minimize observer bias.

### Immunohistochemical examinations

Sections collected onto polylysine-coated slides were immunostained for haptoglobin (Anti-Haptoglobin antibody [EPR22856-212](ab256454),1/100 dilution) and CD31 (Anti-CD31 antibody [EPR17259](ab182981), 1/100 dilution) using the streptavidin-biotin technique. Both primary and secondary antibodies were purchased from Abcam (Cambridge, UK). Following a 60-minute incubation period with primary antibodies, sections were immunohistochemically stained using biotinylated secondary antibodies and streptavidin-alkaline phosphatase conjugate. EXPOSE Mouse and Rabbit Specific HRP/DAB Detection IHC kit -ab80436 was used as the secondary antibody, and diaminobenzidine served as the chromogen (DAB). For the negative controls, the primary antiserum phase was replaced with the antigen dilution solution. Blinded samples were used for all tests. The Database Manual Cell Sens Life Science Imaging Software System (Olympus Co., Tokyo, Japan) was used for microphotography and morphometric analysis.

All antibodies used (Abcam) were validated for rat tissue cross-reactivity and immunohistochemical specificity by the manufacturer. Negative control experiments confirmed the specificity of staining.

Immunoreactive areas were semi-quantitatively evaluated using ImageJ software. Immunopositivity was expressed as a percentage of positively stained area per total field. At least five random fields per section were analyzed at ×400 magnification.

### Reverse transcription-polymerase chain reaction (RT-qPCR)

Using the manufacturer’s protocol, RNA was isolated from homogenized tissues with the GeneAll RiboEx (TM) RNA Isolation Kit (GeneAll Biotechnology, Seoul, Korea). The amount and purity of the RNAs obtained were measured using the BioSpec-nano nanodrop device (Shimadzu Ltd., Kyoto, Japan). 1 µg RNA was used for cDNA synthesis. cDNA synthesis was performed using the A.B.T. ™ cDNA Synthesis Kit (Atlas Biotechnology, Turkey) in a thermal cycler according to the manufacturer›s protocol. Primer designs were made by detecting specific mRNA sequences and testing possible primer sequences using the NCBI website. The sequences of the primers used are shown in Table 1. Expression levels of genes were measured in a Biorad CFX96 (California/USA) real-time PCR instrument using A.B.T.™ cDNA Synthesis Kit (Atlas Biotechnology/ Turkey). In the study, the GAPDH gene was used as a housekeeping gene. The reaction mixture was prepared according to the manufacturer›s protocol to a final volume of 20 µl. The resulting reaction mixture was placed in a real-time qPCR device, with thermal cycling performed according to the kit manufacturer›s protocol. Each sample was analyzed in three replicates. PCR conditions, initial denaturation at 95 ^°^C for 300 sec. 1 cycle, denaturation at 95 ^°^C 15 sec., and annealing/extension at 56 ^°^C 30 sec. were applied to 40 cycles. Relative mRNA levels were calculated by applying the 2^- ΔΔCt^ formula to the normalized results.

### Statistical analysis

The results were analyzed for normality distribution, and since all data were normally distributed, one-way ANOVA was used, followed by Tukey’s multiple comparison test. Statistical analysis was performed using GraphPad Prism version 8.0 software (San Diego, California, USA). Differences were considered significant for *P*<0.05. All results are expressed as mean±SD.

## Results

### Oxidative stress markers

**Figure 3:**
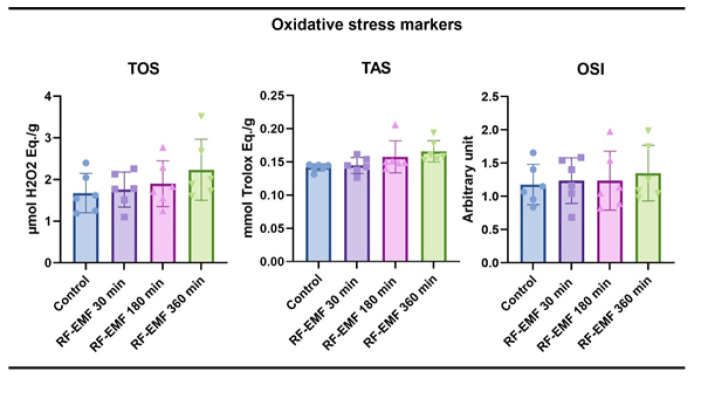
Oxidative stress parameters in Wistar Albino rat brain tissue

When the oxidative stress parameters were analyzed, no statistical significance was found between the groups for any of the parameters. It was found that RF-EMF application did not trigger oxidative stress in brain tissue during the application of RF-EMF at three different times (Figure 3).

### Histopathological findings

**Figure 4:**
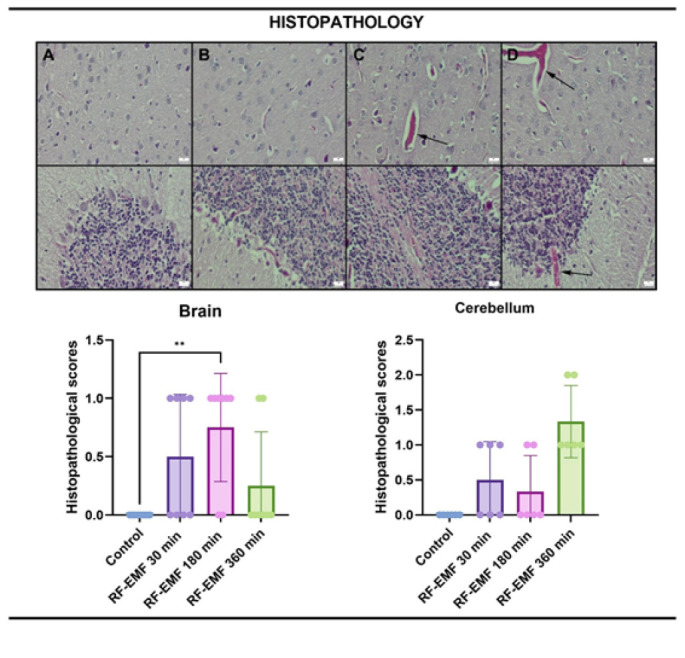
Representative histological sections of the cerebral cortex (upper row) and cerebellum (lower row) from Wistar Albino rats, stained with H&E

During the microscopical examination of the brains and cerebellums, there were no lesions in the control group. The other groups showed slight hyperemia and edema related to the dosage of the exposure (Figure 4).

### Immunohistochemical findings

**Figure 5:**
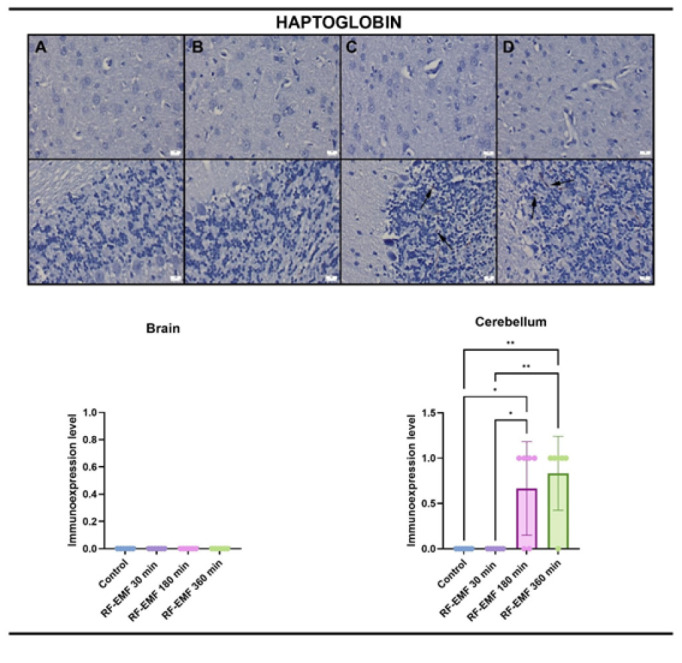
Haptoglobin immunostaining in the cerebral cortex (upper row) and cerebellum (lower row) of Wistar Albino rats

**Figure 6:**
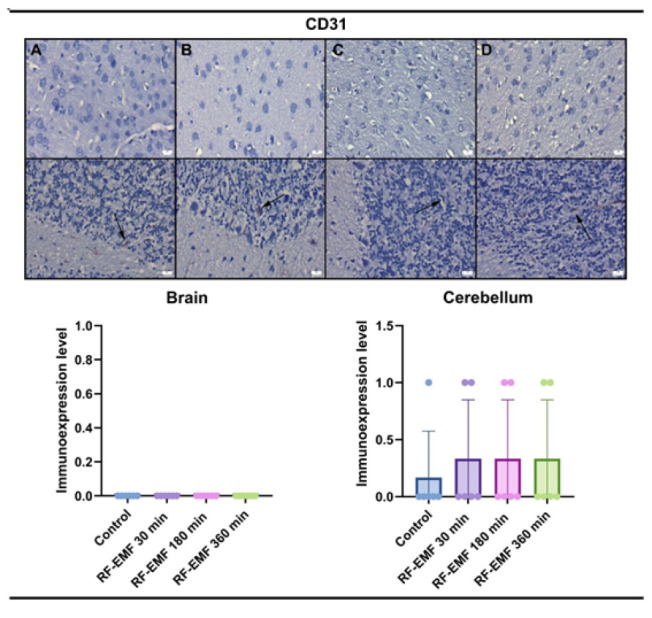
CD31 immunoreactivity in the cerebral cortex (upper row) and cerebellum (lower row) of Wistar Albino rats

At the examination of haptoglobin immunostained slides, only slight expressions were noticed in the cerebellums in the 180-minute and 360-minute exposure groups. No expression was noticed in the other groups (Figures 5, 6).

CD31 immunohistochemical findings revealed negative expressions in the brains in all groups, while slight expressions were observed in the cerebellums. There were no differences between the groups.

### RT-qPCR results

**Figure 7:**
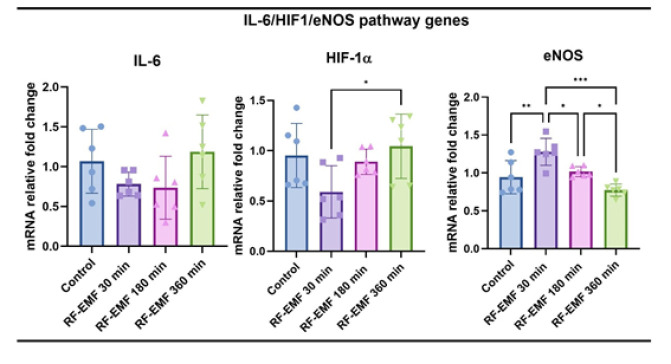
Relative mRNA expression levels of IL-6, HIF-1α, and eNOS in the brain tissue of Wistar Albino rats

No significant difference was observed between groups in terms of IL-6 levels, which is both an acute phase reactant and an activator of the IL-6/HIF-1α/eNOS pathway. Although IL-6 expression did not reach statistical significance, a slight upward trend was observed at 360 min exposure, potentially indicating a subthreshold inflammatory activation. When HIF-1α levels were examined, it was determined that RF-EMF applied for 360 min increased more than the 30-minute group. The NO-producing enzyme eNOS expression was highest in the RF-EMF 30 min (*P*<0.01) group and increased significantly compared to the control group. While eNOS levels were found to be significantly decreased compared to the RF-EMF 30 min group, a significant difference was detected between these two groups (*P*<0.05)(Figure 7).

**Figure 8:**
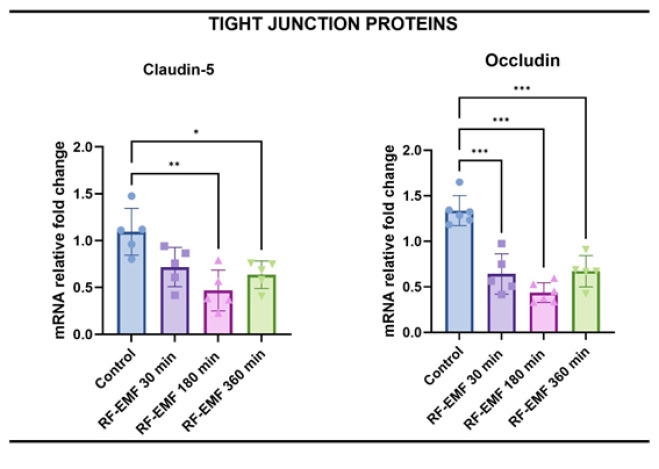
Relative mRNA expression levels of tight junction proteins claudin-1 and occludin in the brain tissue of Wistar Albino rats

When claudin-1 and occludin levels, which are indicators of BBB permeability, are examined, claudin-1 expression decreased in all three groups. In comparison, it was found to be significant in the RF-EMF 180 min and RF-EMF 360 min groups (*P*<0.01 for both). Occludin levels were found to be statistically decreased in all three groups compared to the control group (*P*<0.001 for all)(Figure 8).

## Discussion

The BBB serves as a dynamic and selective interface that protects the CNS from harmful agents while maintaining homeostasis. However, this protective role can become a therapeutic obstacle in diseases requiring drug penetration into the brain parenchyma. Therefore, the ability to transiently and non-invasively modulate BBB permeability has attracted increasing scientific attention (19). In this context, the present study demonstrates that acute exposure to a 27.12 MHz RF-EMF for 30 min significantly modulates BBB permeability by up-regulating eNOS expression and down-regulating occludin, without inducing inflammation or oxidative stress.

In our experimental setup, 30-, 180-, and 360-minute exposures were selected to explore the temporal dynamics of BBB modulation. Our results clearly show that 30-minute RF-EMF exposure leads to a significant increase in eNOS expression. This finding aligns with prior evidence suggesting that RF-EMF enhances endothelial function and tissue perfusion through NO-dependent pathways (14). Notably, no histological damage or inflammation was observed in this group, suggesting that short-term RF-EMF can transiently open the BBB in a safe and controlled manner.

In contrast, the 180- and 360-minute RF-EMF exposures induced histopathological changes such as hyperemia and edema, particularly in cerebellar tissues, indicating a possible inflammatory response. Although IL-6 expression did not reach statistical significance, its upward trend in the 360-minute group supports the presence of low-grade inflammation. Moreover, HIF-1α expression was significantly elevated with increasing exposure duration, reflecting hypoxic stress or reactive metabolic compensation. These findings suggest that while longer RF-EMF exposures can further disrupt BBB integrity, they may do so at the cost of tissue stress and vascular reactivity.

Interestingly, eNOS expression declined in the 180- and 360-minute groups, contrary to the 30-minute result. One plausible explanation is the depletion of L-arginine, the substrate for NO synthesis. Previous studies have shown that prolonged NO synthesis can deplete intracellular arginine pools, particularly under conditions of metabolic stress or inflammation (11). This substrate-limiting mechanism might explain the biphasic eNOS response observed here. Nevertheless, this hypothesis remains speculative and warrants future biochemical validation through arginine quantification or eNOS activity assays.

The tight junction proteins claudin-1 and occludin are fundamental to maintaining BBB integrity. Their down-regulation in the 180- and 360-minute groups aligns with the observed histological findings, suggesting a structural compromise of the barrier. Notably, occludin levels were significantly reduced in all exposure groups, including the 30-minute group. While occludin down-regulation typically indicates increased permeability, the absence of inflammatory or oxidative markers in this group implies that the effect may be reversible and functionally selective. This supports the potential of 30-minute RF-EMF exposure as a therapeutic window for transient BBB opening.

The expression of CD31, a marker of endothelial junction stability and vascular remodeling, remained unchanged across all groups, except for minimal expression in cerebellar tissues. This suggests that RF-EMF-induced BBB modulation does not involve angiogenesis or endothelial proliferation, at least within the exposure durations tested. Similarly, haptoglobin expression, a marker of hemorrhagic stress, was only minimally increased in long-duration groups and absent in 30-minute exposure, further indicating the non-damaging nature of short-term RF-EMF.

Oxidative stress analysis revealed stable TAS, TOS, and OSI levels across all groups, reinforcing the conclusion that RF-EMF, particularly at shorter durations, does not provoke redox imbalance. This contrasts with many other BBB modulation techniques, such as focused ultrasound, which are associated with transient oxidative bursts or microbubble-induced damage (20). Therefore, RF-EMF appears to offer a safer profile for clinical translation, especially in protocols requiring repeated applications.

Compared to focused ultrasound or pulsed magnetic fields, RF-EMF at 27.12 MHz presents advantages such as field uniformity, deeper tissue penetration, and minimal thermal effects at low power densities. Furthermore, unlike pharmacological BBB disruptors (e.g., mannitol or bradykinin analogs), RF-EMF does not induce systemic hypotension or osmotic shifts. These properties make it particularly attractive for localized, on-demand BBB modulation in conditions like brain tumors, epilepsy, or CNS infections.

Despite these promising findings, certain limitations should be acknowledged. The exclusive use of female rats may limit generalizability due to known sex-related differences in endothelial function and BBB response. Additionally, the relatively small sample size and lack of functional assays (e.g., Evans blue permeability or tracer imaging) prevent direct quantification of BBB leakage. Future studies should incorporate both sexes, larger cohorts, and functional permeability assays to comprehensively validate the effects of RF-EMF.

Moreover, the absence of long-term follow-up prevents us from assessing the reversibility of BBB opening or potential delayed tissue damage. Investigating whether the BBB re-seals within hours or days post-exposure would be essential before clinical translation. The integration of drug delivery trials combining RF-EMF exposure with chemotherapeutics or neuroprotectants would also reveal the functional utility of this approach.

In summary, this study reveals that 30-minute exposure to 27.12 MHz RF-EMF significantly enhances BBB permeability via eNOS up-regulation and occludin down-regulation without inducing histological, oxidative, or inflammatory damage. Longer exposures, while further disrupting barrier structure, may introduce tissue stress and should be approached cautiously. These results provide foundational insight into RF-EMF as a non-invasive BBB modulation tool, with significant implications for drug delivery and neurotherapeutic strategies.

RF-EMF: Radiofrequency electromagnetic field, IL-6: Interleukin-6, HIF-1α: Hypoxia inducible factor-1 alpha, eNOS: Endothelial nitric oxide synthase, Total oxidant status, TAS: Total antioxidant status, OSI: Oxidative stress index, IHK: Immunohistochemical, HP: Histopathological

Octagonal geometry is appropriate for using the PCB antenna as an applicator in the operating frequency

**Table 1:** Primary sequences, product size, and accession numbers of genes analyzed in Wistar Albino rats

Genes	**Primary sequence**	**Product size**	**Accession number**
eNOS	F: GGTTGACCAAGGCAAACCAC	247 bp	NM_021838.2
	R: CCTAATACCACAGCCGGAGG		
**Occludin**	F: TCACTGTGTGACCTGTCTTGG	217 bp	NM_031329.3
	R: ACTGGGCTGGATGCCAATTT		
**Claudin 1**	F: ACTGTGGATGTCCTGCGTTT	127 bp	NM_031699.3
	R: CCCCAGCAGGATGCCAATTA		
**IL-6**	F: CACAAGTCCGGAGAGGAGAC	168 bp	NM_012589.2
	R: ACAGTGCATCATCGCTGTTC		
**HIF-1α**	F: GCAACTAGGAACCCGAACCA	251 bp	NM_024359.2
	R: TCGACGTTCGGAACTCATCC		
**GAPDH (Housekeeping)**	F: AGGTTGTCTCCTGTGACTTC	130 bp	NM_017008.4
	R: CTGTTGCTGTAGCCATATTC		

F: Forward, R: Reverse, GAPDH: glyceraldehyde-3-phosphate dehydrogenase, IL-6: Interleukin-6, HIF-1α: Hypoxia inducible factor-1 alpha, eNOS: Endothelial nitric oxide synthase

Data presented as the mean±standard deviation. The oxidative stress markers were compared between groups using one-way ANOVA (*post-hoc* Tukey’s test). *P<0.05; **P<0.01; ***P<0.001

RF-EMF: Radiofrequency electromagnetic field, TOS: Total oxidant status, TAS: Total antioxidant status, OSI: Oxidative stress index

(A) Normal architecture in the control group. (B) No marked change in 30-min RF-EMF group. (C) Moderate hyperemia in the 180-min group (arrow). (D) Severe hyperemia and edema in the 360-min group (arrows). Scale bars=50 μm

(A) No staining in the control. (B) Negative in a 30-minute group. (C) Slight positivity in the cerebellum of the 180-min group (arrow). (D) Slight positivity in 360-min group cerebellum (arrow). Scale bars=50 μm. IHC analysis used the streptavidin-biotin DAB protocol. * *P<*0.05; ** *P<*0.01; *** *P<*0.001

(A) Control, (B) 30-min, (C) 180-min, (D) 360-min exposure. No immunostaining observed in cerebral cortex; minimal expression in cerebellar vessels in all groups (arrows). Scale bars=50 μm. * *P<*0.05; ** *P<*0.01; *** *P<*0.001

Data are mean±SD (n=3/group). eNOS expression significantly increased in the 30-min RF-EMF group compared to the control. One-way ANOVA followed by Tukey’s test. *P<0.05, **P<0.01, ***P<0.001

Claudin-1 significantly decreased in 180- and 360-min groups, while occludin expression was reduced across all RF-EMF exposure durations. Data are presented as mean±SD (n=3). *P<0.05, **P<0.01, ***P<0.001

## Conclusion

In this study, 27.12 MHz RF-EMF exposure for 30 min significantly modulated BBB permeability by up-regulating eNOS and down-regulating occludin, without inducing oxidative or inflammatory damage. Longer exposures (180 and 360 min) caused structural disruption, increased HIF-1α levels, and minor haptoglobin expression, indicating tissue stress. These results suggest that short-term RF-EMF may offer a safe and effective non-invasive strategy for transient BBB opening.

However, the study is limited by the use of only female rats, a small sample size, and the absence of functional permeability or long-term follow-up data. Future research should evaluate sex-related responses, reversibility of BBB modulation, and therapeutic co-application with pharmacological agents. Nonetheless, our findings lay essential groundwork for the development of RF-EMF-based BBB targeting technologies in translational neuroscience and drug delivery.

## Data Availability

All relevant data generated or analyzed during this study are included in this article. Further inquiries can be directed to the corresponding author.

## Declaration

We have not utilized any AI tools or technologies in the preparation of this manuscript.
